# Fluid-solid coupling numerical simulation of the effects of different doses of verapamil on cancellous bone in type 2 diabetic rats

**DOI:** 10.1186/s12891-024-07235-1

**Published:** 2024-02-09

**Authors:** Xiaodan Wu, He Gong, Xiaorong Hu

**Affiliations:** Key Laboratory of Biomechanics and Mechanobiology, Ministry of Education, Beijing Advanced Innovation Center for Biomedical Engineering, School of Biological Science and Medical Engineering, Beihang University, Beijing, 100083 China

**Keywords:** Type 2 diabetes, Verapamil, Trabecular bone, Fluid-solid coupling simulation, Fluid shear stress

## Abstract

**Background:**

The purpose of this study was to investigate the effects of four different doses of verapamil on the mechanical behaviors of solid and the characteristics of fluid flow in cancellous bone of distal femur of type 2 diabetes rats under dynamic external load.

**Methods:**

Based on the micro-CT images, the finite element models of cancellous bones and fluids at distal femurs of rats in control group, diabetes group, treatment groups VER 4, VER 12, VER 24, and VER 48 (verapamil doses of 4, 12, 24, and 48 mg/kg/day, respectively) were constructed. A sinusoidal time-varying displacement load with an amplitude of 0.8 μm and a period of 1s was applied to the upper surface of the solid region. Then, fluid-solid coupling numerical simulation method was used to analyze the magnitudes and distributions of von Mises stress, flow velocity, and fluid shear stress of cancellous bone models in each group.

**Results:**

The results for mean values of von Mises stress, flow velocity and FSS (t = 0.25s) were as follows: their values in control group were lower than those in diabetes group; the three parameters varied with the dose of verapamil; in the four treatment groups, the values of VER 48 group were the lowest, they were the closest to control group, and they were smaller than diabetes group. Among the four treatment groups, VER 48 group had the highest proportion of the nodes with FSS = 1-3 Pa on the surface of cancellous bone, and more areas in VER 48 group were subjected to fluid shear stress of 1-3 Pa for more than half of the time.

**Conclusion:**

It could be seen that among the four treatment groups, osteoblasts on the cancellous bone surface in the highest dose group (VER 48 group) were more easily activated by mechanical loading, and the treatment effect was the best. This study might help in understanding the mechanism of verapamil’s effect on the bone of type 2 diabetes mellitus, and provide theoretical guidance for the selection of verapamil dose in the clinical treatment of type 2 diabetes mellitus.

## Introduction

Diabetes can be divided into type 1 and type 2 diabetes, of which type 2 diabetes (T2DM) is the most common, accounting for more than 90% of the total number of diabetes patients [1]. Type 2 diabetes can not only cause vascular damage and endanger the heart, kidney, brain, eyes and peripheral nerves, but also reduce bone formation and delay bone healing, leading to an increased risk of fracture [2–4]. Our previous experimental study has shown that verapamil treatment can improve blood glucose, bone mass, bone microstructure parameters and mechanical properties in type 2 diabetic rats [5]. In order to understand the bone formation and bone resorption of cancellous bone in rats with type 2 diabetes before and after verapamil treatment, it is necessary to understand their bone remodeling process, which is an important indicator to evaluate the effect of drugs on the treatment of type 2 diabetes.

Bone remodeling is mainly composed of osteoblasts-dominated bone formation and osteoclasts-dominated bone resorption. Cancellous bone is the most active area of bone remodeling, which is composed of trabeculae and marrow. There are various types of cells in bone marrow, including blood cells, osteoblasts, osteoclasts, adipocytes, and mesenchymal stem cells [6–8]. Bone marrow is a viscous fluid, and its viscosity is generated by intercellular interactions [9]. When the bone is subjected to external loads, the deformation of the bone matrix can cause fluid flow in the cancellous bone system [10–13]. Studies have shown that when bone perceives mechanical stimulation, fluid shear stress will be generated, which will cause osteoblasts or osteoclasts on the surface of cancellous bone to respond, and ultimately regulate bone remodeling to adapt bone to its mechanical environment [14–18]. Some scholars have carried out studies on the effects of fluid shear stress on osteoblasts and osteoclasts: it has been found that fluid shear stress can regulate the proliferation and differentiation of osteoblasts in vitro, and the mode and extent of regulation are different depending on the magnitude and duration of the force [9, 19, 20]. Under normal physiological load, the FSS level of 1-3 Pa can induce the biological response of osteoblasts in vitro [19]; fluid-solid coupling numerical simulation was carried out on the constructed flat flow chamber and cell finite element models, and it was found that fluid flow could regulate the calcium response and migration of osteoclast precursor cells and mature osteoclasts, and osteoclast precursors RAW264.7 could sense the gradient of FSS and migrate to the low FSS regions [21, 22]. Because of the complexity of cancellous bone structure, the solid and fluid excitations felt by cells in vivo and in vitro are different, so it is necessary to clarify the magnitudes and distributions of von Mises stress, flow velocity and fluid shear stress in cancellous bones, which is of great significance for further understanding the regulatory mechanism of verapamil therapy on bone remodeling in type 2 diabetes rats.

Due to the lack of in vivo measurement experimental techniques, fluid-solid coupling numerical simulation is an effective method for studying the spatial distribution of fluid flow in bone. Scholars have conducted numerous studies on bones using fluid-solid coupling numerical simulation methods. For example, an ideal dental cancellous bone model containing pore structure was constructed, and the distributions of parameters such as fluid flow, pressure, and fluid shear stress were calculated using fluid-solid coupling numerical simulation method by some scholars [23]; Metzger et al. obtained a real geometric model of cancellous bone of pig femur through three-dimensional reconstruction, and also used fluid-solid coupling numerical simulation to study the distributions of fluid shear stress and pressure in the trabecular pore space [24]; Zhao et al. carried out fluid-solid coupling numerical simulation on cancellous bone of rat femur, and found that flow velocity, fluid shear stress and pressure gradient increased linearly with the increase of loading frequency [18]; Sandino et al. obtained the distributions of von Mises stress, octahedral strain, strain energy density, flow velocity and pore pressure in bone and bone marrow phase of human tibia through numerical simulation [25]; Tian et al. established a fluid-solid coupling finite element model for the L4-L5 segment of degenerative lumbar spine with lumbar disc herniation, and verified the effectiveness of the model, providing theoretical guidance for the clinical treatment of lumbar disc herniation in the later stage [26]. In summary, establishing a fluid-solid coupling finite element model of bone can accurately simulate the mechanical response of human or animal bones, and obtain fluid-related mechanical parameters that are difficult to obtain in vitro.

Most of the relevant literature on fluid-solid coupling numerical simulation is to obtain solid and fluid mechanical parameters through normal human and animal studies, but there is no fluid-solid coupling numerical simulation on the effect of different doses of verapamil therapy on cancellous bone in type 2 diabetes rats. In this study, solid and fluid mechanical parameters in cancellous bones of rats in control group, diabetes group and verapamil treatment groups under dynamic external load were analyzed by fluid-solid coupling numerical simulation, which provided basic data for elucidating the regulatory mechanism of bone remodeling, was of great significance for further understanding the mechanism of the effect of verapamil on bone of type 2 diabetes mellitus, and provided theoretical guidance for the selection of verapamil dosage in the clinical treatment of type 2 diabetes mellitus. In addition, it is also of great significance for the follow-up study of verapamil combined with exercise in the treatment of type 2 diabetes. The exercise method that is more in line with the force required for bone rehabilitation should be chosen.

## Materials and methods

### Bone samples and micro-CT imaging

The 18 cancellous bone samples of distal femurs were obtained from our previous study on the effects of verapamil on bone mass, microstructure and mechanical properties in type 2 diabetic rats, which had previously received ethical approval [5]. The samples were obtained from the right femurs of six groups of 25-week-old SD male rats, i.e., control group (CON), diabetes group (T2DM), and treatment groups VER 4, VER 12, VER 24, and VER 48 (verapamil doses of 4, 12, 24, and 48 mg/kg/day, respectively), with 3 samples in each group. The feeding methods of each group were as follows. Control group was 7-week-old healthy SD male rats, which were fed normal diet until 25 weeks old; after 1 week of adaptive feeding, the remaining 7-week-old healthy SD male rats were fed with high-fat and high-carbohydrate chow for 4 weeks and then fasted for 12-16 h (without water). Then, the newly configured STZ was injected into the abdominal cavity once, with a dose of 35 mg/kg [27, 28]. After 7 days, blood glucose of the tail vein in the fasting state was measured, and the blood glucose concentration of the rats greater than 16.7mmol/L was type 2 diabetes rats that were successfully modeled [29, 30], and the model rats were divided into diabetes group and four treatment groups. For treatment groups, verapamil hydrochloride tablets were dissolved in 0.5% sodium carboxymethyl cellulose to prepare suspension. Treatment groups VER 4, VER 12, VER 24 and VER 48 were administrated intragastatically at 4, 12, 24 and 48 mg/kg/day twice a day for 12 weeks, respectively; except for the fasting process, the rats in treatment groups were continuously fed with high-fat and high-carbohydrate chow.

After the left and right femoral muscles and soft tissues were removed, the left and right femurs of the rats were respectively placed in different centrifuge tubes with normal saline and stored in a -20℃ refrigerator for future use. The right femur samples of rats were selected and thawed naturally. The femoral regions were scanned using Micro-CT (Skyscan1076, Bruker, Luxemburg, Belgium). The spatial resolution was set to 18 μm, the voltage was 70 kV, the current was 140 mA, the scanning power was 10 W, the filter plate was selected as 0.5 mm aluminum plate, the rotation angle was 180° and 2 pieces were scanned every 0.6° to obtain the scanning images. The micro-CT images were reconstructed using Mimics Research 16.0 software (Materialise, Leuven, Belgium).

### The establishment of finite element models

**Fig. 1:**
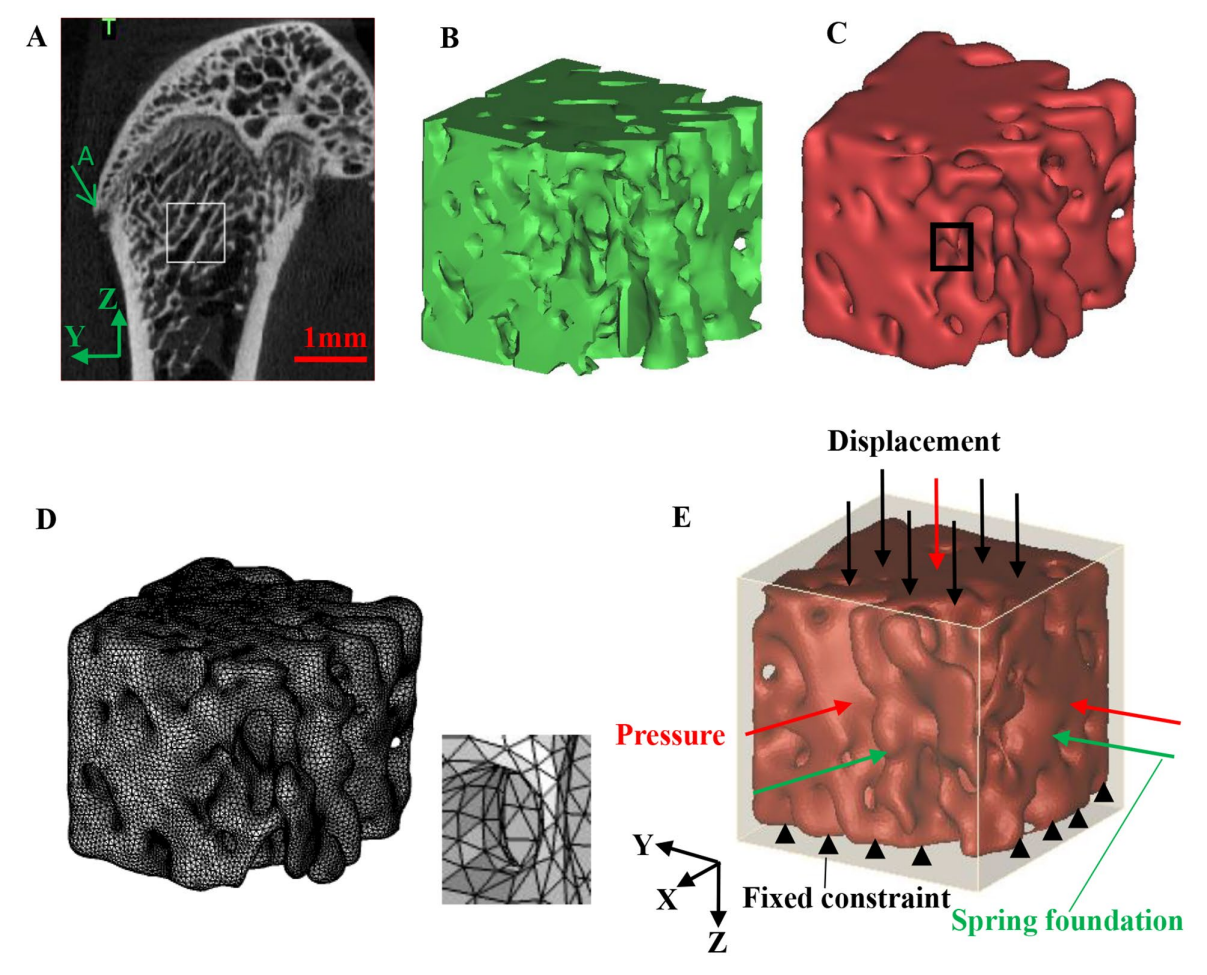
Establishment of fluid-solid coupling model and boundary conditions. **A**. Micro-CT image of rat femur in control group, **B**. Cancellous bone model in the marked area of Fig. 1A initially established by Mimics software, **C**. Cancellous bone model optimized by Geomagic Studio software, **D**. Finite element models of cancellous bone in the marked area of Fig. 1A and the black wireframe in Fig. 1C, **E**. Boundary conditions applied to cancellous bone and fluid models. The black arrows represent displacement loading, the red arrows show fluid pressure, the green arrows indicate elastic foundation, and the black arrow heads denote fixed constraint

The cuboidal region of 0.8 × 0.8 × 0.8mm^3^ was selected from the cancellous bone of the distal femur as the solid domain (Fig. 1A-D), and the 0.86 × 0.86 × 0.86mm^3^ cube around the cancellous bone was selected as the fluid domain filled with bone marrow fluid (Fig. 1E). All cancellous bone samples were selected in the same position. Figure 1A was taken as the first image along the X direction when cube model of cancellous bone was selected. The leftmost intersection point between the outer contour curve of the femur at the top and the epiphyseal line in Fig. 1A was defined as A. The region was selected with a length of 0.8 mm from point A along the negative direction of the Y axis at 1 mm, and a length of 0.8 mm along the negative direction of the Z axis at 1.3 mm from the highest point of the epiphyseal line, and a length of 0.8 mm along the negative direction of the X axis. The solid and fluid models established above were imported from Mimics software into Geomagic Studio software (Geomagic Inc, USA) for optimization processing. Under the “polygon” module of Geomagic Studio software, the “sandpaper” function combined with “nail removal”, “relaxation”, “quick smoothing”, “noise reduction” and other functions were used to smooth the surfaces. The strength value in the “sandpaper” function, and the smoothness level values in the “nail removal”, “relaxation” and “noise reduction” functions were set to be relatively small with the values of 3, 10, 4, and 1, respectively, so that the solid and fluid models resulting from the optimized processing were not distorted. Through the above operations, the inner and outer surfaces of cancellous bone and fluid models became smooth. Cancellous bone model optimized by Geomagic Studio software is shown in Fig. 1C. The specific method for meshing cancellous bone (including thinner cancellous bone sites) and fluid models was as follows. The solid and fluid models after optimization in Geomagic Studio software were imported into 3-matic Research 12.0 software (Materialise, Leuven, Belgium) for tetrahedral meshing. Firstly, the “inspection measure” function was used under the “remeshing” module to check the quality of the triangular plates. Secondly, in the “remeshing” module, the “auto remesh” function was used to divide the surface mesh of the solid and fluid models, and the surface mesh was divided into triangles, and the “maximum triangle edge” was set as 0.01 mm and the “shape quality” was set as 0.09. Then the “reduce” function was used under the “fix” module to optimize the quality of the triangular plates. Finally, “create volume mesh” from the “remeshing” toolbar was selected to generate the volume mesh, and the element size was 0.01 mm. The cancellous bone model after meshing was shown in Fig. 1D. The total numbers of elements for all solid and fluid models ranged from 64,720 to 155,148 and from 120,458 to 261,124, respectively.

Fluid-solid interaction (FSI) analysis was used to investigate von Mises stress distribution in bone matrix and fluid shear stress (FSS) distribution in bone marrow. Cancellous bone was set as a uniform and isotropic linear elastic material. In our previous study on the effects of four different doses of verapamil treatment on blood glucose, bone mass, bone microstructure parameters and mechanical properties of type 2 diabetic rats, transverse and longitudinal elastic moduli of trabecular bones of femoral heads in each group were obtained by nano-indentation test [5]. The elastic moduli of CON, T2DM, VER 4, VER 12, VER 24 and VER 48 models were the weighted average of the corresponding transverse and longitudinal elastic moduli of trabecular bones of femoral heads in each group [31], which were 15.08GPa, 11.28GPa, 12.00GPa, 12.60GPa, 13.63GPa and 14.43GPa, respectively. Poisson’s ratio was set at 0.3 [32]. Bone marrow was set as a Newtonian fluid with a density of 0.95 g/cm^3^ and a dynamic viscosity coefficient of 85.5 Pa·s [32].

### Boundary conditions

A fixed constraint was applied to the bottom of the solid region, and a sinusoidal time-varying displacement load was applied to the upper surface of the solid domain as follows:

1 $$u\left( {\rm{t}} \right) = \frac{B}{2}\left\{ {\sin \left[ {2\pi \left( {{\rm{t - }}0.25} \right)} \right]} \right. + \left. 1 \right\}$$

Where the amplitude *B* is set to 0.8 μm [33].

Spring foundations were applied to the remaining four sides of the solid domain to simulate the mechanical effects of the surrounding cancellous bone on the simulated region [9]. The expression is as follows:

2 $${T_{\rm{t}}} = - k\left[ {{U_{solid}}\left( {\rm{t}} \right) - {U_0}} \right]$$

Where the spring constant *k* is set to 16 × 10^9^N/mm^2^ [34], *U*_*solid*_ and *U*_0_ are the current and initial displacement of the solid surface, respectively.

The six faces of the fluid region were open boundaries that allowed fluid to enter and exit freely. A normal pressure was applied to each of the six surfaces of the fluid. The expression is as follows:

3 $${P_X},{P_Y}\,{\rm{or}}\,{{\rm{P}}_{\rm{Z}}} = \frac{C}{2}\left\{ {{\rm{sin}}\left[ {2\pi \left( {t - 0.25} \right)} \right]} \right. + \left. 1 \right\}$$

Where the amplitude *C* is set to 667 Pa [18].

### Data analysis

Transient FSI analyses of all models were performed using COMSOL Multiphysics software (COMSOL, Stockholm, Sweden) [33] to obtain von Mises stress distributions of cancellous bones, flow velocity distributions of fluid in cancellous bones and fluid shear stress distributions on the surface of cancellous bones. To avoid boundary effects, datas in the 0.7 × 0.7 × 0.7mm^3^ sub-regions at the center of the cancellous samples were selected for analysis. For all cancellous bone samples (18 samples) of rats in control group, diabetes group and four treatment groups, FSS of surface nodes were expressed as medians and quartiles respectively, and proportions of the nodes with FSS in different ranges were expressed as medians respectively. The distribution ranges of von Mises stress, flow velocity and fluid shear stress in typical models were expressed as means ± standard deviations. Due to the relatively small number of rats in each group for calculating mean FSS of cancellous bone surface, and not all data of the same parameter were normally distributed, non-parametric test (Kruskal-Wallis test of K independent samples) was used to evaluate differences among groups. Post-hoc analysis for non-parametric test was then used to determine the difference in mean FSS on the surface of cancellous bone between each two groups of samples. Origin 2018 software (OriginLab Inc, USA) was used for statistical analysis, and *P* < 0.05 was considered statistically significant.

## Results

### Von Mises stress distribution of cancellous bone of distal femur in each group

**Fig. 2:**
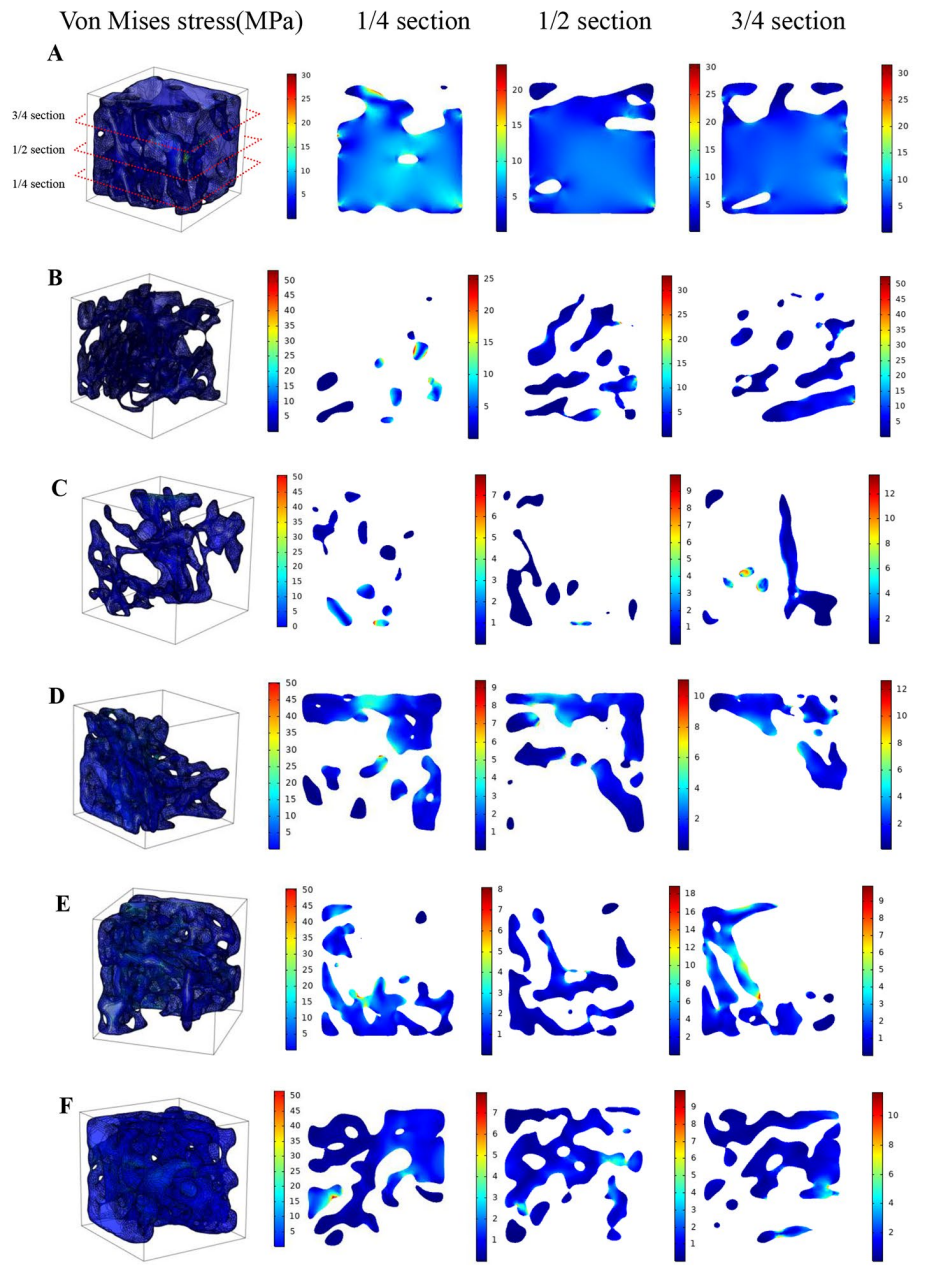
Von Mises stress distribution of cancellous bone of distal femur in each group and in different sections along the loading direction (t = 0.25s). (One representative model was selected from control group, diabetes group and four treatment groups, respectively.) **A**. CON, **B**. T2DM, **C**. VER 4, **D**. VER 12, **E**. VER 24, **F**. VER 48

Von Mises stress distributions of a typical cancellous bone of distal femur selected from control group, diabetes group, and four treatment groups (t = 0.25s) were shown in Fig. 2, respectively. Because fluid shear stress value was the largest at t = 0.25s = T/4 in a loading cycle, the results for the moment of 0.25s were listed specifically in this paper. The means ± standard deviations of von Mises stresses of cancellous bones in typical models of control group, diabetes group and treatment groups VER 4, VER 12, VER 24 and VER 48 were 1.41 ± 1.68 MPa, 1.50 ± 2.21 MPa, 2.45 ± 7.10 MPa, 1.93 ± 2.24 MPa, 1.99 ± 2.25 MPa, and 1.13 ± 1.55 MPa, respectively. For control group, diabetes group and treatment groups VER 4 and VER 12, the maximum von Mises stress was found on the side of cancellous bone. The results for mean of von Mises stress were as follows: control group was smaller than diabetes group; VER 48 group was the lowest in the four treatment groups, it was the closest to control group, and it was smaller than diabetes group. In addition, in order to observe solid von Mises stress in the bone tissue more clearly, von Mises stress distributions in the 1/4, 1/2 and 3/4 sections of cancellous bones of distal femurs along the loading direction in control group, diabetes group and four treatment groups were shown in Fig. 2 (t = 0.25s). Except for VER 24 group, in control group, diabetes group, and treatment groups VER 4, VER 12, and VER 48, high von Mises stress mainly appeared in the upper region, which mainly bore the load under PL.

### Velocity distribution of fluid in cancellous bone of distal femur in each group

**Fig. 3:**
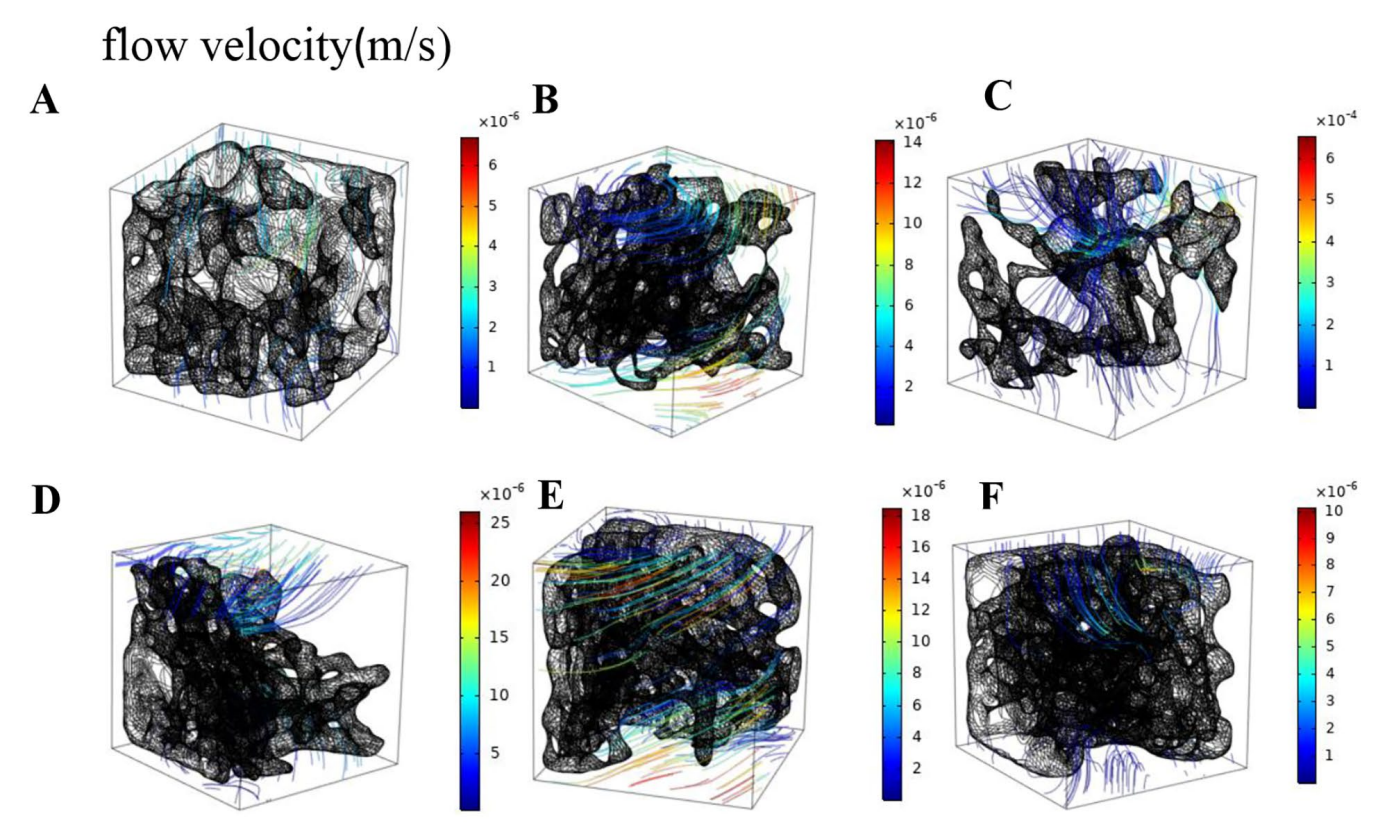
Flow velocity distribution of fluid in cancellous bone of distal femur in each group (t = 0.25s). (One representative model was selected from control group, diabetes group and four treatment groups, respectively.) **A**. CON, **B**. T2DM, **C**. VER 4, **D**. VER 12, **E**. VER 24, **F**. VER 48

Flow velocity distributions of fluid in a typical distal femoral cancellous bone selected from control group, diabetes group, and four treatment groups (t = 0.25s) were shown in Fig. 3, respectively. The means ± standard deviations of flow velocities of cancellous bones in typical models of control group, diabetes group, treatment groups VER 4, VER 12, VER 24 and VER 48 were (0.08 ± 0.40)×10^− 6^m/s, (0.45 ± 1.65)×10^− 6^m/s, (0.12 ± 0.51)×10^− 4^m/s, (0.49 ± 2.13)×10^− 6^m/s, (0.57 ± 2.15)×10^− 6^m/s, and (0.09 ± 0.45)×10^− 6^m/s, respectively. Flow velocities of cancellous bones in control group, treatment groups VER 4, VER 12 and VER 48 near the solid loading surface (PL) were higher, and the fluid in the domain mainly flowed along the direction of PL loading. In diabetes group, cancellous bone had a higher flow velocity near the medial femur. Mean of flow velocity in control group was lower than that in diabetes group; in the four treatment groups, mean of flow velocity of VER 48 group was the lowest, it was the closest to control group, and it was smaller than diabetes group.

### Fluid shear stress distribution of cancellous bone of distal femur in each group

**Fig. 4:**
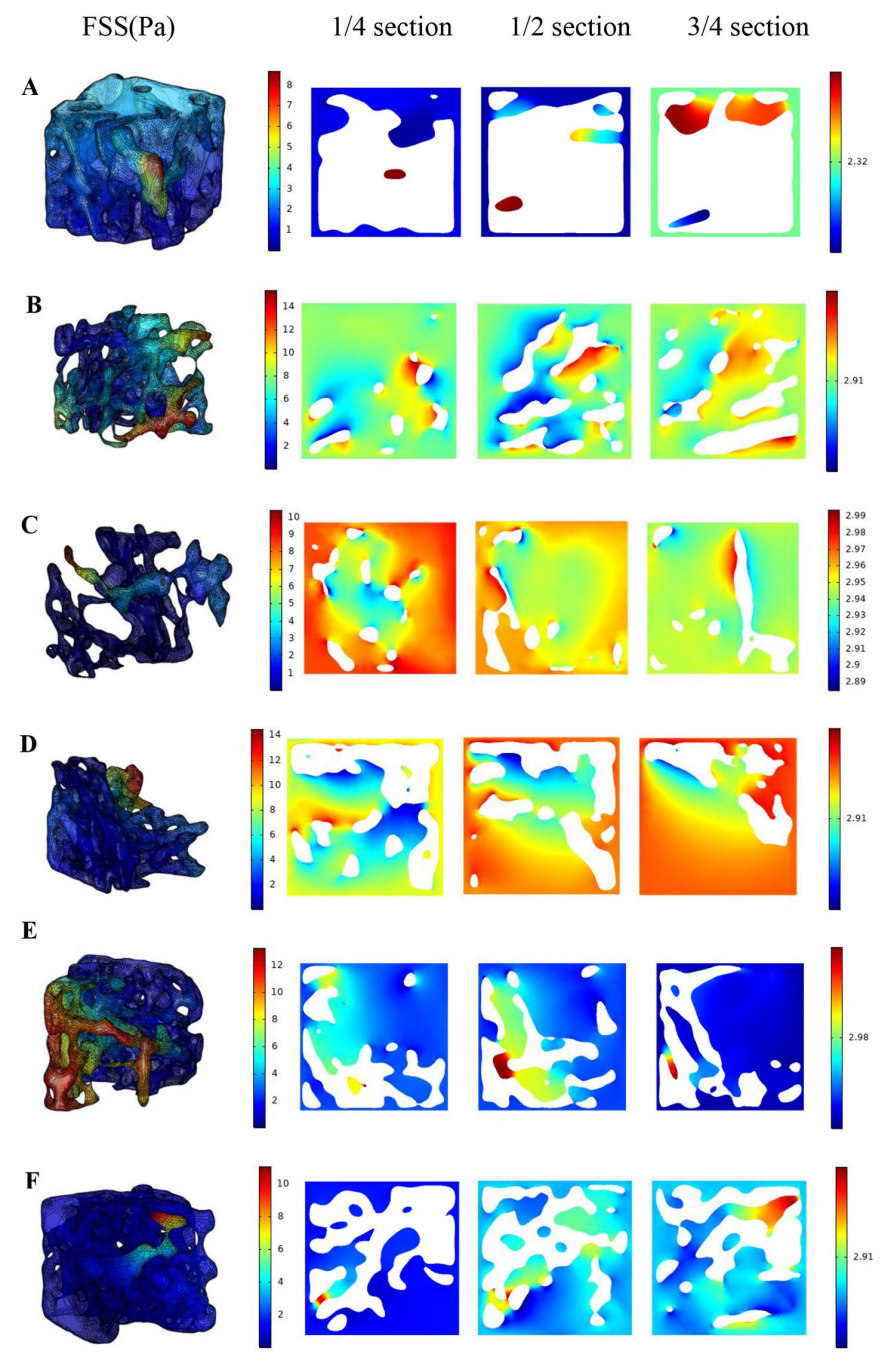
Fluid shear stress distribution of cancellous bone of distal femur in each group and in different sections along the loading direction (t = 0.25s). (One representative model was selected from control group, diabetes group and four treatment groups, respectively.) **A**. CON, **B**. T2DM, **C**. VER 4, **D**. VER 12, **E**. VER 24, **F**. VER 48

Fluid shear stress distributions of a typical cancellous bone of distal femur selected from control group, diabetes group, and four treatment groups (t = 0.25s) were shown in Fig. 4, respectively. The means ± standard deviations of FSS of cancellous bones in typical models of control group, diabetes group, treatment groups VER 4, VER 12, VER 24 and VER 48 were 1.60 ± 0.89 Pa, 3.89 ± 2.95 Pa, 4.06 ± 1.73 Pa, 2.86 ± 2.33 Pa, 3.97 ± 3.46 Pa, and 2.18 ± 0.75 Pa, respectively. The results for mean value of FSS were as follows: its value in control group was lower than that in diabetes group; in the four treatment groups, the value of VER 48 group was the lowest, it was the closest to control group, and it was smaller than diabetes group. In control group, cancellous bone had a larger FSS in the upper region, while in diabetes group, cancellous bone had a larger FSS near the medial femur. In addition, in order to observe the fluid flow in the bone tissue more clearly, fluid shear stress distributions in the 1/4, 1/2 and 3/4 sections of cancellous bones of distal femurs along the loading direction in control group, diabetes group and four treatment groups were shown in Fig. 4 (t = 0.25s). There were differences in the size and quantity of pores in different sections. In cancellous bone of diabetes group, there were fewer internal pores in the 1/4 section, and more large pores in the 1/2 and 3/4 sections. In control group, diabetes group and four treatment groups, high shear stress mainly appeared at the interface between solid and fluid in the internal pores of cancellous bone.

### Surface fluid shear stress of cancellous bone of distal femur in each group

**Fig. 5:**
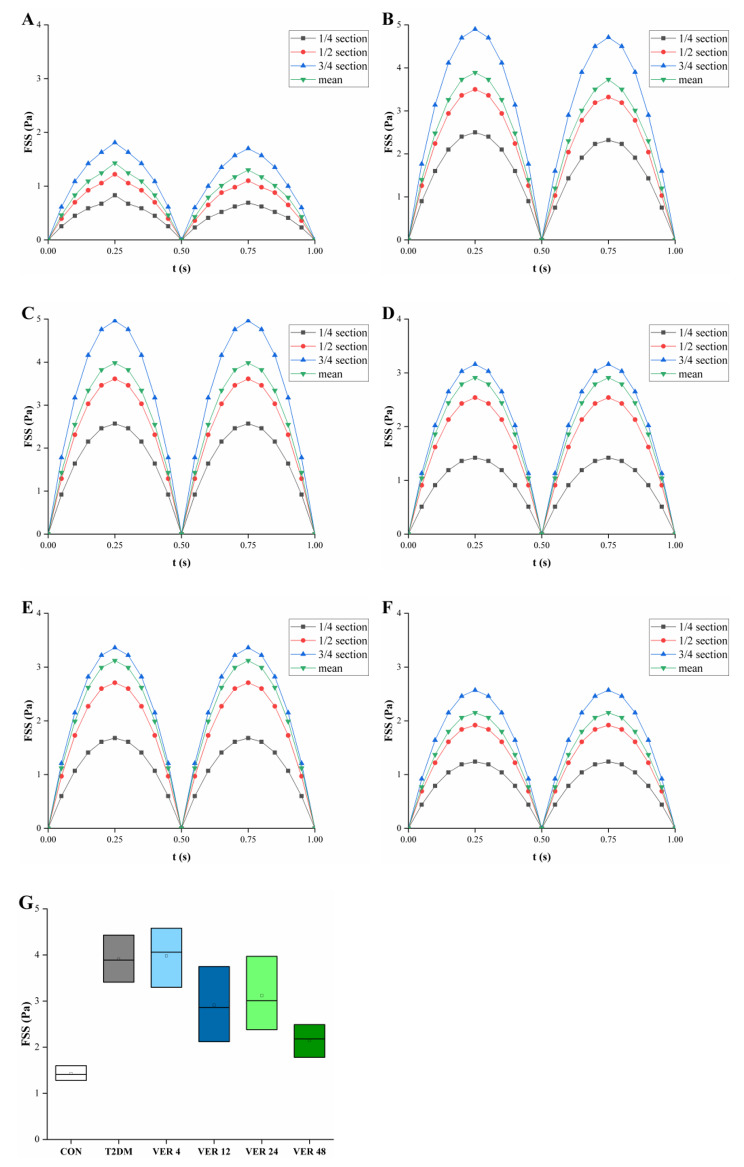
Changes in mean of surface fluid shear stress of all cancellous bone samples in different groups, mean of surface fluid shear stress in 1/4, 1/2 and 3/4 sections of cancellous bone along the loading direction over time, and mean of fluid shear stress. **A**. CON, **B**. T2DM, **C**. VER 4, **D**. VER 12, **E**. VER 24, **F**. VER 48, **G**. t = 0.25s

Surface FSS values of all cancellous bone samples in control group, diabetes group and four treatment groups were quantitatively compared in Fig. 5A-F. Surface FSS values of cancellous bones reached its maximum values at 1/4 moment of each loading cycle. It could be seen that whether means of surface FSS of all cancellous bone samples in each group or means of surface FSS of cancellous bones in 1/4, 1/2 and 3/4 sections along the loading direction, surface FSS values of cancellous bones in diabetes group were greater than those in control group, indicating that the level of FSS on the surface of cancellous bone in diabetes group was higher than that in control group. The peaks of mean FSS were VER 4, T2DM, VER 24, VER 12, VER 48, CON in descending order. Mean of surface FSS of cancellous bone in each group decreased gradually in 3/4, 1/2 and 1/4 sections along the loading direction. As could be seen from Fig. 5G, when t = 0.25s, the medians of means of surface FSS of all cancellous bone samples in diabetes group and four treatment groups were higher than those in control group, but there were no significant differences (*p* > 0.05). There were no significant differences in the median of mean FSS of cancellous bone surface among all groups (*p* > 0.05); among the four treatment groups, the median of mean FSS of cancellous bone surface in treatment group VER 48 was the lowest (*p* > 0.05).

**Fig. 6:**
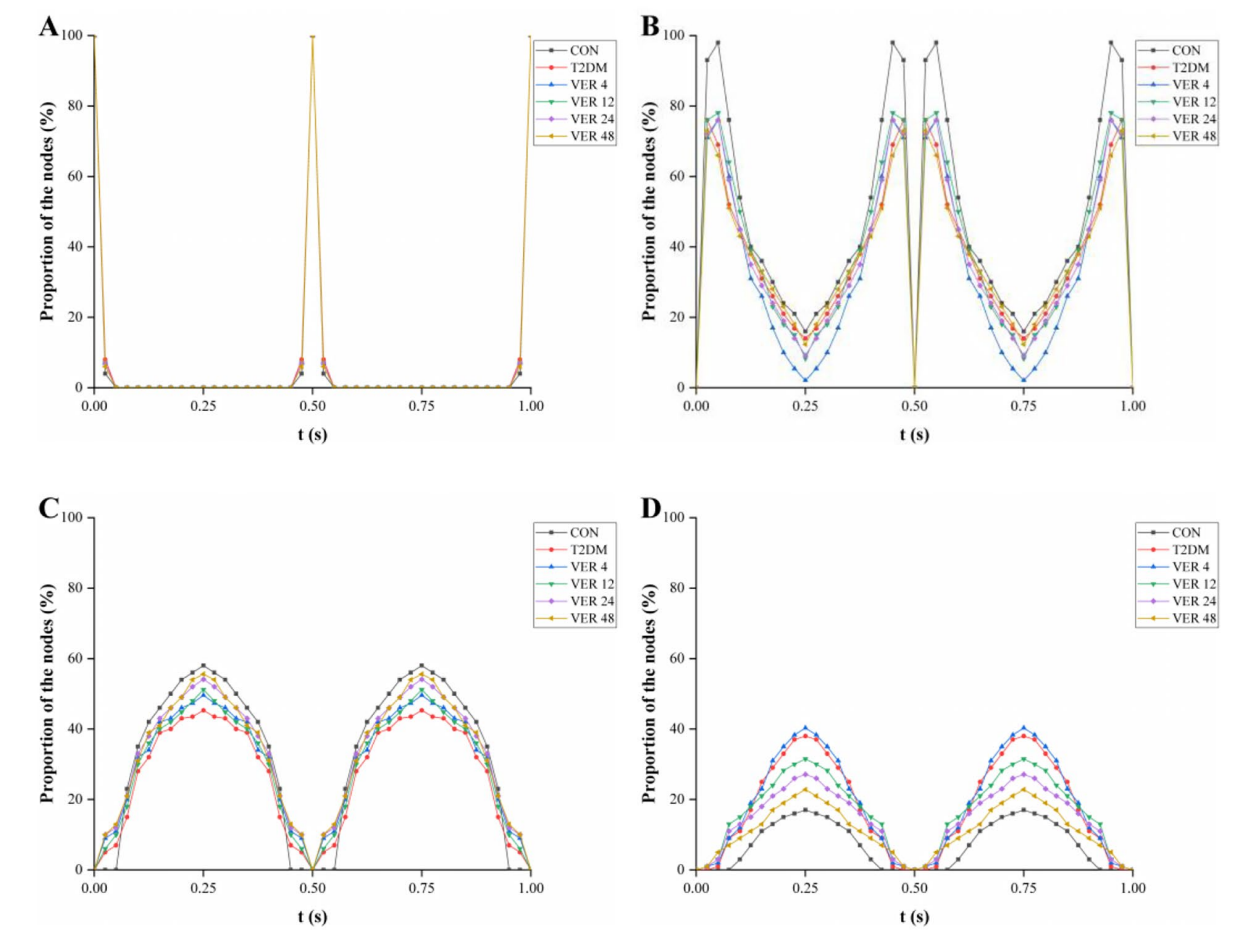
Proportion of the nodes with FSS in different range over loading duration in each group. (Each point in the Figure is the median obtained by statistical analysis of proportion of the nodes in all samples in the group.) **A**. FSS < 0.1 Pa, **B**. FSS = 0.1-1 Pa, **C**. FSS = 1-3 Pa, **D**. FSS > 3 Pa

In Fig. 6, a statistical comparison was made on proportion of the nodes with FSS < 0.1 Pa, FSS = 0.1-1 Pa, FSS = 1-3 Pa, and FSS > 3 Pa on the surface of all cancellous bone samples in control group, diabetes group and four treatment groups. For most of the loading cycle, there was almost no FSS < 0.1 Pa area on the cancellous bone surface. 42%, 32%, 34%, 36%, 38%, 39% of the cancellous bone surface in control group, diabetes group and treatment groups VER 4, VER 12, VER 24 and VER 48 were stimulated by FSS of 1-3 Pa for more than 1/2 time, respectively. The results for median proportion of the nodes were as follows: when t = 0.25s, proportions of the nodes with FSS = 1-3 Pa on the surface of cancellous bones were CON, VER 48, VER 24, VER 12, VER 4, and T2DM in descending order; as the dosage of verapamil increased, proportion of the nodes with FSS = 1-3 Pa on the surface of cancellous bone gradually increased; among the four treatment groups, VER 48 group had the highest proportion of the nodes with FSS = 1-3 Pa on the surface of cancellous bone. The results of bone cell culture experiment in vitro indicated that osteoblasts would produce biological response when the applied fluid shear stress was between 1 and 3 Pa [19]. Therefore, osteoblasts on the surface of cancellous bones in control group were more easily activated by mechanical loading than those in diabetes group, which were conducive to starting the process of bone formation. Among the four treatment groups, osteoblasts on the surface of cancellous bones in VER 48 group were more easily activated by mechanical loading, thus facilitating the initiation of bone formation.

## Discussion

In order to further understand the regulatory mechanism of verapamil treatment on bone remodeling in type 2 diabetes rats, it is necessary to study the fluid flow in cancellous bone of femur in rats under mechanical stimulation. However, it is difficult to directly observe or measure the micro-mechanical environment in which the bone marrow is located. Fluid-solid coupling numerical simulation method is appropriate in estimating the fluid flow inside the bone caused by bone deformation. There are many factors affecting fluid shear stress, including bone volume fraction, elastic modulus, bone cavity permeability, and loading patterns [9, 24, 35–37]. Through our previous study on the effects of different doses of verapamil on bone mass, bone microstructure and bone mechanical properties of type 2 diabetes rats, it was concluded that the elastic moduli of cancellous bone models in four different doses of verapamil treatment groups were different [5]. So this study focused on the effects of different doses of verapamil on magnitudes and distributions of FSS, flow velocity, and solid von Mises stress under normal physiological load.

**Fig. 7:**
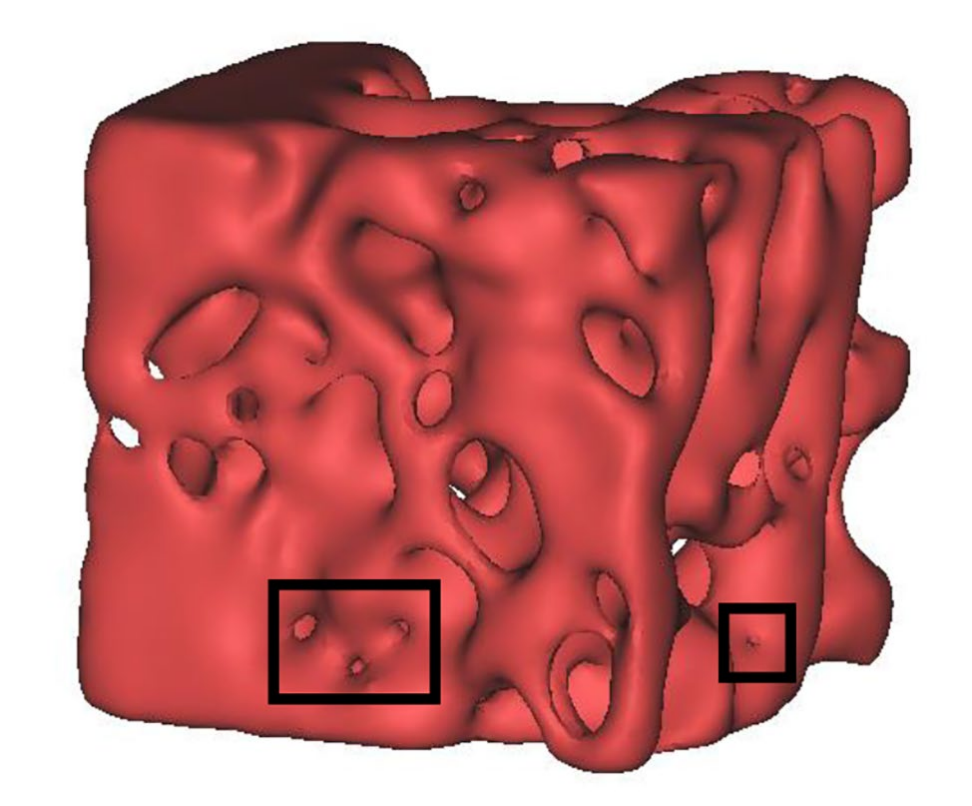
Typical cancellous bone model in VER 48 group. (There were relatively narrow pores in the black wire frames.)

In our study, the results of solid von Mises stress, flow velocity and FSS were similar to those reported by Zhao et al., showing the same order of magnitude [18]. It was found in this study that except for VER 24 group, high von Mises stress in the other five groups mainly appeared in the upper region; in control group, diabetes group and four treatment groups, high shear stress mainly appeared in the fluid-solid interface of the internal pores in the upper region of cancellous bone; the fluid of cancellous bone in control group, treatment groups VER 4, VER 12, and VER 48 had a higher flow velocity near the solid loading surface. This indicated that the upper region of cancellous bone was the main bearing region, which also explained why trabeculae in cancellous bone mainly grew along the direction of principal stress to a certain extent. The results of this study were consistent with the previously reported relationship between von Mises stress and FSS: Hambli et al. concluded that solid von Mises stress was positively correlated with FSS [38]; Li et al. conducted fluid-solid coupling numerical simulation on cancelous bone of mouse femur and found a positive correlation between von Mises stress in solid matrix and FSS in fluid field at the fluid-solid interface [9]. Among them, flow velocities of fluid in cancellous bones of the typical models were expressed as the means ± standard deviations, and the results showed that the standard deviations were greater than the mean values, indicating that the data were relatively discrete and flow velocities varied in a large range. The reason for the high dispersion might be that there were relatively narrow pores in cancellous bone structure (the black wire frames in Fig. 7).

In this study, the results for the two parameters of mean FSS in cancellous bone of distal femur and mean flow velocity of its internal fluid (t = 0.25s) were as follows: their values in control group were lower than those in diabetes group; among the four treatment groups, the values of VER 48 group were the lowest, they were the closest to control group, and they were smaller than diabetes group. The essential reason was that FSS and flow velocity were negatively correlated with bone volume fraction. In this study, bone volume fraction of each group was characterized as follows: control group was higher than diabetes group; among the four treatment groups, VER 48 group was the highest, it was the closest to control group, and it was larger than diabetes group (Bone volume fractions of typical models in CON, T2DM, treatment groups VER 4, VER 12, VER 24 and VER 48 were calculated using CTAn software (CTAn, Bruker, Luxemburg, Belgium), and their values were 76.8%, 23.7%, 12.96%, 28.85%, 32.16% and 49.06%, respectively). Therefore, FSS and flow velocity of each group were characterized as follows: control group was smaller than diabetes group; among the four treatment groups, VER 48 group was the lowest, it was the closest to control group, and it was smaller than diabetes group. This was consistent with the previously reported relationship between FSS and flow velocity with bone volume fraction: a previous study applied fluid-solid coupling numerical simulation method to analyze the femoral cancellous bone model of pig, and concluded that FSS would decrease with the increase of bone volume fraction [24]; some scholars conducted fluid-solid coupling analyses on the constructed ideal models of cancellous bone and bone marrow, and found that with the decrease of bone volume fraction, FSS and flow velocity increased [23].

This study showed that the dose of verapamil affected the magnitude and distribution of fluid shear stress in the cancellous bone of distal femur in rats under dynamic external load. Numerous studies concluded that bone tissue optimized its structure to adapt to the mechanical loading environment in which it was placed [39, 40]. In the absence of mechanical stimulation, bone mineral loss would occur, while motion and mechanical stimulation would produce fluid shear stress, causing osteoblasts or osteoclasts on the surface of cancellous bone to respond, thus increasing or decreasing the mineral content in bone [14–17, 41, 42]. Trabeculae in cancellous bone grew mainly in the direction of principal stress, thus bearing external loads in the most efficient structural form. The results of bone cell culture experiment in vitro showed that osteoclasts migrated to low-FSS regions less than 0.1 Pa [21, 43]; when the applied fluid shear stress was between 1 and 3 Pa, osteoblasts would produce biological response [19, 20]; FSS greater than 4 Pa could lead to osteoblast apoptosis [9]. This study indicated that when t = 0.25s, proportions of the nodes with FSS = 1-3 Pa on the surface of cancellous bones were CON, VER 48, VER 24, VER 12, VER 4, and T2DM in descending order; with the increase of verapamil dose, proportion of the nodes with FSS = 1-3 Pa on the surface of cancellous bone gradually increased; among the four treatment groups, VER 48 group had the highest proportion of the nodes with FSS = 1-3 Pa on the surface of cancellous bone. In control group, diabetes group and treatment groups VER 4, VER 12, VER 24, and VER 48, 42%, 32%, 34%, 36%, 38% and 39% of the cancellous bone surface were stimulated by FSS of 1-3 Pa for more than 1/2 time, respectively. Among the four treatment groups, the highest dose group VER 48 had more areas subjected to 1 to 3 Pa of fluid shear stress for more than half of the time. Therefore, osteoblasts on the surface of cancellous bone in control group were more easily activated by mechanical loading than those in diabetes group. Among the four treatment groups, osteoblasts on the cancellous bone surface in VER 48 group were more easily activated by mechanical loading. These results indicated that the effects of verapamil on the magnitude and distribution of FSS on the surface were dose-dependent. In this study, verapamil at a dose of 48 mg/kg/day had the best therapeutic effect, providing guidance for the future use of verapamil in the treatment of type 2 diabetes patients.

Our study has some limitations. In this paper, it was assumed that cancellous bone was homogeneous and isotropic linear elastic material, ignoring the viscoelasticity of bone marrow; considering the time and resources required for calculation, it was difficult to calculate the entire femur model. Fluid-solid coupling analysis was performed only on a small cube area of cancellous bone selected on the distal femur of control group, diabetes group and four treatment groups with different verapamil doses, separately. Despite these limitations, this study enabled us to understand the effects of different doses of verapamil treatment on the magnitudes and distributions of FSS, flow velocity, and solid von Mises stress, providing basic data for elucidating the regulatory mechanism of bone remodeling.

## Conclusions

In conclusion, this study obtained solid and fluid mechanical parameters in femoral cancellous bones of rats in control group, diabetes group and four treatment groups with different verapamil doses under dynamic external load through fluid-solid coupling numerical simulation analysis. The results showed that the magnitudes and spatial distributions of FSS, solid von Mises stress and flow velocity varied with verapamil dose. Among the four treatment groups, the highest dose (48 mg/kg/day) of verapamil was more conducive to bone formation and had the best therapeutic effect. These results can help us to better understand the mechanism of verapamil’s effect on the bone of type 2 diabetes mellitus, and provide theoretical guidance for the selection of verapamil dose in the clinical treatment of type 2 diabetes mellitus.

## Data Availability

The datasets used during the present study are available from the corresponding author on reasonable request.
